# The Hyperlipidaemic Drug Fenofibrate Significantly Reduces Infection by SARS-CoV-2 in Cell Culture Models

**DOI:** 10.3389/fphar.2021.660490

**Published:** 2021-08-06

**Authors:** Scott P. Davies, Courtney J. Mycroft-West, Isabel Pagani, Harriet J. Hill, Yen-Hsi Chen, Richard Karlsson, Ieva Bagdonaite, Scott E. Guimond, Zania Stamataki, Marcelo Andrade De Lima, Jeremy E. Turnbull, Zhang Yang, Elisa Vicenzi, Mark A. Skidmore, Farhat L. Khanim, Alan Richardson

**Affiliations:** ^1^ Institute for Immunology and Immunotherapy, University of Birmingham, Birmingham, United Kingdom; ^2^ Molecular and Structural Bioscience, School of Life Sciences, Keele University, Staffordshire, United Kingdom; ^3^ Viral Pathogenesis and Biosafety Unit, San Raffaele Scientific Institute Via Olgettina, Milano, Italy; ^4^ Copenhagen Center for Glycomics, Department of Cellular and Molecular Medicine, Faculty of Health Sciences, University of Copenhagen, Copenhagen, Denmark; ^5^ Department of Biochemistry and Systems Biology, Institute of Systems, Molecular and Integrative Biology, University of Liverpool, Liverpool, United Kingdom; ^6^ School of Biomedical Sciences, Institute for Clinical Sciences, University of Birmingham, Birmingham, United Kingdom; ^7^ School of Pharmacy and Bioengineering, Keele University, Staffordshire, United Kingdom

**Keywords:** fenofibrate, fibrate, SARS-CoV-2, COVID-19, ACE2

## Abstract

The severe acute respiratory syndrome coronavirus-2 (SARS-CoV-2) pandemic has caused a significant number of fatalities and worldwide disruption. To identify drugs to repurpose to treat SARS-CoV-2 infections, we established a screen to measure the dimerization of angiotensin-converting enzyme 2 (ACE2), the primary receptor for the virus. This screen identified fenofibric acid, the active metabolite of fenofibrate. Fenofibric acid also destabilized the receptor-binding domain (RBD) of the viral spike protein and inhibited RBD binding to ACE2 in enzyme-linked immunosorbent assay (ELISA) and whole cell-binding assays. Fenofibrate and fenofibric acid were tested by two independent laboratories measuring infection of cultured Vero cells using two different SARS-CoV-2 isolates. In both settings at drug concentrations, which are clinically achievable, fenofibrate and fenofibric acid reduced viral infection by up to 70%. Together with its extensive history of clinical use and its relatively good safety profile, this study identifies fenofibrate as a potential therapeutic agent requiring an urgent clinical evaluation to treat SARS-CoV-2 infection.

## Introduction

Severe acute respiratory syndrome coronavirus-2 (SARS-CoV-2) is responsible for a pandemic, which has cost over 1.9 million lives worldwide so far (Dhama et al., 2020; World Health Organization, 2020; Wu et al., 2020). The emergence of new virus variants with higher transmissibility rates is seeing rapid increases in infection rates and deaths across the world. Several vaccines have undergone accelerated approval and are being rolled out worldwide (Baden et al., 2021; Voysey et al., 2021). While the clinical data are very promising, the vaccines are not recommended or suitable in all patient groups, e.g., children, those with hyperimmune disorders, and those using immunosuppressants (Meo et al., 2021), and with the global spread of viral variants of concern, e.g., Alpha-B.1.1.7, Beta-B.1.351, Gamma-P.1, and Delta-B.1.617.2, it is presently unclear whether the current vaccines will offer sufficient protection to emerging strains (Meo et al., 2021). While in a few countries vaccination programs are progressing at speed, vaccine uptake rates are variable and for most low middle-income countries, significant proportions of the population are unlikely to be vaccinated until 2022. Furthermore, while vaccination has been shown to reduce infection rates and severity of disease, we are as yet unsure of the strength and duration of the response. Therapies are still urgently needed to manage COVID-19 patients who develop symptoms and/or require hospitalization.

The virus gains entry to human cells by the receptor-binding domain (RBD) of the viral spike protein binding to angiotensin-converting enzyme 2 (ACE2) on human cells (Clausen et al., 2020; Hoffmann et al., 2020). Although other receptors of the virus have been identified (Cantuti-Castelvetri et al., 2020; Daly et al., 2020), drugs that block virus binding to ACE2 may substantially reduce virus uptake, thereby reducing/relieving symptoms in patients with an active infection or reduce transmission of the virus to uninfected individuals.

While the rapid escalation of the SARS-CoV-2 epidemic leaves insufficient time to develop new drugs via traditional pipelines, drug repurposing offers an expedited and attractive alternative. Drugs which are repurposed are available for immediate clinical use and their pharmacokinetic and safety profiles are usually well described. This has already proven true, with the identification that dexamethasone reduces mortality of SARS-CoV-2 patients (RECOVERY Collaborative Group et al., 2021) and remdesivir decreases the time needed for patients to recover from infection (Beigel et al., 2020). In these cases, although the drugs are technically being repurposed, their use still depends on the drug’s recognized mechanism of action. It is less obvious which drugs might have a novel mechanism of action and interfere with SARS-CoV-2 binding and cellular entry mediated by ACE2. To this end, we recently developed an assay to measure the viral spike protein’s RBD binding to ACE2 (Lima et al., 2021).

Structural studies have shown that ACE2 is a dimer and that there may be multiple spike RBDs interacting with each ACE2 dimer (Yan et al., 2020). Molecular dynamic simulations have suggested considerable flexibility in ACE2 and this might allow multiple ACE2 dimers to bind to each spike trimer (Barros et al., 2021). If this were to be the case, the dimerization of ACE2 would lead to multiple contacts with each spike trimer, increasing the avidity of the binding. Alternatively, dimerization of ACE2 might sterically hinder the protomers from binding to the spike protein. It therefore seems reasonable that the extent of ACE2 dimerization might affect the avidity of RBD binding. Furthermore, dimerization has been shown to affect the internalization of other receptors. For example, dimerization of EGF or FGF receptors promotes their endocytosis (Wang et al., 2005; Opalinski et al., 2017) and different mechanisms of internalization may exist for monomeric and dimeric GH receptors (Gent et al., 2002). This led to the hypothesis that drugs that altered dimerization of ACE2 might affect viral infection by endocytosis. In order to test this hypothesis, we developed an assay to measure dimerization of ACE2, making use of the NanoBIT protein interaction system (Dixon et al., 2016). This is based on a modified luciferase (NanoLuc) which has been split into two catalytically incomplete components, LgBIT and SmBIT, that must bind together to form an active luciferase. LgBIT and SmBIT associate with low affinity but when fused to other proteins that interact with each other, colocalization of the fusion proteins allows an active luciferase to be formed (Dixon et al., 2016). Here, we have used this system to measure dimerization of ACE2 and screened a library of approved drugs (FMC Library (Khanim et al., 2011)) using an unsupervised approach to identify drug candidates for repurposing. Our experiments demonstrated that fenofibric acid (Supplementary Figure S1), the active metabolite of the oral hyperlipidaemic drug fenofibrate, apparently induced ACE2 dimerization and destabilized the spike RBD inhibiting binding of spike RBD to ACE2. Importantly and as hypothesized, fenofibrate-induced changes in RBD-ACE2 interactions correlated with significantly lower infection levels (< 60%) and viral release in cell culture models using live SARS-CoV-2. Our data combined with unpublished data from other groups and the existing clinical knowledge of fenofibrate identify it as a strong candidate for treating SARS-CoV-2 infections.

## Materials and Methods

### Materials

The plasmid pcDNA3 encoding ACE2 was obtained from GenScript (OHu20260); the plasmid encoding prolactin (PRL) was obtained from Sino Biological (HG10275-CY). OptiMEM and Lipofectamine 2000 were obtained from Thermo Fisher Scientific. NanoBIT and high affinity binary interaction technology (HiBIT) detection reagents, flexicloning transfer systems (C8820 and C9320), and NanoBIT starter kit (N2015) were obtained from Promega. This provides protein kinase A regulatory (PRKAR2) and catalytic subunits (PRKACA) with NanoBIT reporters which serve as a positive interaction control. Anti-His antibody was from Thermo Fisher Scientific (37-2900) and anti-FLAG from Cell Signaling Technology (#2368). The plasmid pcDNA3 encoding ACE2-FLAG was obtained from GenScript (OHu20260) and pcDNA3 encoding ACE2-SBP-6xHis was obtained from Thermo Fisher Scientific. S1 protein was obtained from the National Institute of Biological Standards (United Kingdom).

### Molecular Biology

Full-length ACE2 was amplified by PCR using primers (forward GAC​CGC​GAT​CGC​CAT​GTC​AAG​CTC​TTC​CTG​GCT​CCT​TCT; reverse GAT​GGT​TTA​AAC​AAA​GGA​GGT​CTG​AAC​ATC​ATC​AGT​G) to introduce a 5’ Sgf1 restriction site immediately prior to the start codon and a Pme1 restriction site directly after the codon encoding the last phenylalanine residue. The PCR product was digested with Flexiblend (Sgf1 and Pme1), gel purified, and ligated into pF4ACMV before verifying by sequencing. The insert was subsequently transferred into either pFC34K (encoding LgBIT) or pFC36K (encoding SmBIT) using the C-terminal flexicloning system to generate C-terminal fusion proteins. The tagged ORFs are then expressed under the control of a herpes simplex virus (HSV) thymidine kinase promoter. Atg5 and Atg16 tagged with NanoBIT reporters were generated by the authors previously (Crowley et al., 2020).

### NanoBIT and HiBIT Assays

HEK-293 cells were grown in DMEM supplemented with 10% (v/v) fetal calf serum (FCS) and penicillin-streptomycin (50 U/ml). For each well of 384 plates, 1.25 μL of OptiMEM containing 10 ng/μL of each of pFC34K ACE2 and pFC36K ACE2 was mixed with an equal volume of OptiMEM containing 8% lipofectamine-2000. After incubating at room temperature for 30 min, the transfection mix was mixed with 10 volumes of well-dispersed HEK-293 cells (300,000 cells/mL) in 10% FCS/DMEM without antibiotics and 25 μL plated per well of white 384-well plates. The two outer rows of the plate were filled with 25 μL media as a humidity barrier. After 48 h, 2.8 μL drug at 10 x the final concentration was added per well and incubated for 1 h. Detection reagent was prepared by mixing per well 6.33 μL of detection reagent buffer, 0.33 μL of the substrate, and 8.34 μL of OptiMEM containing 10 mM Hepes prewarmed to 37°C. 15 μL detection reagent was added per well and gently mixed and luminescence was read every 10 min over 30 min.

To test whether the drugs inhibit NanoLuc directly, HiBIT-RBD was prepared as described previously (Lima et al., 2021) and the drug was added to the desired final concentration and mixed with an equal volume of HiBIT detection reagent and luminescence measured. The results were compared to the luminescence measured using HiBIT-RBD containing DMSO.

To measure whether the drugs inhibited the binding of HiBIT-RBD to ACE2, drugs were tested in the binding assay as previously described on ice (Lima et al., 2021). Alternatively, binding was measured after 20 min at 37°C. For experiments in which the order of addition was varied, either cell expressing ACE2 or HiBIT-RBD cell culture supernatant was incubated at 37°C for 30 min prior to mixing and binding after a further 20 min measured as described above. To control any effects of the drug on cell number, parallel plates were stained with sulforhodamine B (Witham et al., 2007) and the luminiscence measurements were normalized to this.

### Precipitation of ACE2 Complexes

HEK-293 cells were transfected by mixing (for each well of a six-well plate) 0.5 μg of each pcDNA3 ACE2-FLAG and pcDNA3 ACE2-SBP-6xHis in 50 μL OptiMEM. pCMV3 Prolactin (PRL) was used as a negative control in the absence of plasmids encoding ACE2. 50 μL of 8% Lipofectamine-2000 in OptiMEM was added to plasmid DNA and after 30 min incubation, 1 ml of HEK-293 cells (300,000 per ml) was added, and the suspension was plated per well in six-well plates. After 12 h incubation, the cell culture supernatant was gently removed and replaced with fresh DMEM containing 10% FCS. After a further 6 h, the medium was again removed and the cells were lysed in 250 µL RIPA as previously described (Richardson et al., 1997). Lysates were cleared by centrifugation (20,000 g, 10 min, and 4°C), 30 μL saved for analysis, while 200 μL was mixed with 20 µL of streptavidin beads for 2 h at 4°C. The beads were washed twice with RIPA and once with Tris-buffered saline before being separated on a 4–12% SDS-PAGE gel and transferred to PVDF and proteins were detected with anti-FLAG (1/1,000) or anti-His (0.08 μg/ml) antibodies.

### Expression of the Spike S1-Receptor Binding Domain for ELISA

Secreted Spike S1-RBD was produced stably using CHOZN GS−/− cells in suspension employing a plasmid encoding residues 319–591 of 2019-nCoV S (upstream of a C-terminal HRV3C protease cleavage site, mFc tag, and 8xHis Tag, gifted by Jason S. McLellan, University of Texas, Austin), as described by Tree et al. (2020). The coding region of RBD-Fc was subcloned into a modified pCGS3 (Merck/formally known as Sigma-Aldrich) for glutamine selection in CHOZN GS−/− cells. Briefly, a RBD-Fc stable clone was obtained by electroporation with 2 × 10^6^ cells and 5 μg endotoxin-free plasmids using Amaxa kit V and program U24 with Amaxa Nucleofector 2B (Lonza, Switzerland). Electroporated cells were subsequently plated in 96 wells at 500 cells/well in Plating Medium containing 80% EX CELL^®^ CHO Cloning Medium (Cat.no C6366) and EX CELL CHO CD Fusion serum-free media without glutamine. High-expressing clones were scaled up in serum-free media without l-glutamine in 50 ml TPP TubeSpin^®^ shaking Bioreactors (180 rpm, 37°C, and 5% CO2) for RBD-Fc production. A HiTrap Protein G, HP column (GE Healthcare, US), equilibrated in 1x PBS prior to use, was employed to purify the Spike S1-RBD, eluting with glycine (100 mM, pH 2.7). Purity was confirmed using SDS-PAGE with Coomassie stain and quantified using the bicinchoninic acid assay (Thermo Scientific).

### ELISA Measuring RBD-ACE2 Binding

An RBD-ACE2 interaction ELISA was performed as described by Tree *et al.* (2020). Streptavidin (3 μg/ml; Fisher) was precoated onto the surface of 96-well plates (high binding; Greiner) in Na_2_CO_3_ buffer (50 mM; pH 9.6; 1 h; 37°C). Plates were washed 3x (300 μL PBS containing 0.2% w/v Brij35) prior to blocking for 1 h at 37°C with 50 μL PBS, 0.2% w/v Brij35, and 1% w/v casein. After washing 3x with PBS, plates were coated with 50 μL of 100 ng/ml biotin-ACE2 (Sino Biological) in PBS containing 0.2% w/v Brij35 and 1% w/v casein for 1 h at 37°C. Plates were then washed and incubated at room temperature in 50 µL of 5 μg/ml RBD in PBS containing 0.2% w/v Brij35 and 1% w/v casein for 30 min in the presence or absence of test drugs. Plates were incubated (1 h; 37°C) to allow binding before three washes. Bound RBD was detected by incubation (1 h; 37°C) with rabbit anti-SARS-CoV-2 spike RBD (Stratech) (1:2000 v/v) in PBS containing 0.2% w/v Brij35 and 1% w/v casein. Following three further washes, plates were incubated (30 min; at 37°C) with horseradish peroxidase-conjugated donkey anti-rabbit IgG (1:2500 v/v), in PBS containing w/v Brij35 and 1% w/v casein. Plates were washed five times before the addition of 3,3′,5,5′-tetramethylbenzidine substrate, prepared as per manufacturer’s instructions (Sigma-Aldrich). Color development was halted after 10 min by the addition of H_2_SO_4_ (2 M) and quantified at λ_abs_ = 450 nm using a Tecan Infinite M200 Pro multiwell plate reader (Tecan Group). Specific binding was determined by subtracting the absorbance measured in samples lacking ACE2.

### Differential Scanning Fluorimetry

Differential scanning fluorimetry (DSF) was conducted with 1 μg RBD in 40 μL PBS (pH 7.6) with 1.25x SYPRO™ Orange (Invitrogen) and either H_2_O, sodium acetate, or fibrates in 96-well qPCR plates (AB Biosystems). AB Biosystems, StepOne Plus, and qPCR machine with a TAMRA filter were employed to perform melt curve experiments, increasing the temperature by + 0.5°C every 30 s, from 25 to 90°C. First-order differential plots were calculated after smoothing (Savitzky–Golay, nine neighbors, second-order polynomial) using Prism 8 (GraphPad). The peak maxima of the first-order differential plots were determined with MATLAB software (R2018a, MathWorks) and used to calculate the change in T_m_ in the presence of fibrates. Control wells without RBD, but containing sodium acetate or fibrates, were tested to confirm that altered T_m_ values were a result of protein-ligand interactions and not a result of an interaction between the drug and the dye.

### Modified “CETSA” Assay

A modified version of the cellular thermal shift assay (Martinez et al., 2018) was performed in which 200 μL HiBIT-RBD cell culture supernatant (Lima et al., 2021) cleared of cells by centrifugation (150 g, 3 min) was mixed with an equal volume of 460 μM fenofibric acid for 20 min at 37°C for 20 min. 30 μL samples were incubated for 7 min at a temperature ranging from 37 to 70°C, quenched on ice, and then centrifuged (20,000 g, 20 min, and 4°C). Soluble RBD in the supernatant was measured by mixing 10 μL with 10 μL HiBIT detection reagent.

### SARS-CoV-2 Infection Experiments (hCOV-19/England/2/2020 Strain)

Experiments using live SARS-CoV-2 experiments were all performed in approved BSL 3/CL3 facilities by staff trained to work with BSL3/CL3 infectious organisms using standardized safety and decontamination protocols. Vero cells (ATCC^®^ CCL-81) were washed with PBS, dislodged with 0.25% Trypsin-EDTA (Sigma life sciences), and seeded into 96-well imaging plates (Greiner) at a density of 8 × 10^3^/well in culture media (DMEM containing 10% FBS, 1% penicillin and streptomycin, 1% l-glutamine, and 1% non-essential amino acids). The next day, cells were infected with SARS-CoV-2 strain hCOV-19/England/2/2020, isolated by Public Health England (PHE) from the first patient cluster in the United Kingdom on January 29, 2020. Virus stock 10^6^ IU/ml (kind gift from Christine Bruce, PHE) was diluted 1/150 in culture media allowing 25 μL per well. The virus was then diluted further with 25 μL per well of media containing the drugs being evaluated at twice the desired final concentration to give 1x drug and a final virus dilution of 1/300. Cells were then infected with the virus (167 IU/well) and cultured for 24 or 48 h. After the infection period, supernatants were harvested and frozen prior to analysis by qRT-PCR, and cells were fixed in ice-cold methanol. Cells were then blocked in PBS containing 10% FBS and stained with rabbit anti-SARS-CoV-2 spike protein, subunit 1 (The Native Antigen Company), followed by Alexa Fluor 555-conjugated goat anti-rabbit IgG secondary antibody (Invitrogen, Thermo Fisher Scientific). Cell nuclei were stained with Hoechst 33,342 (Thermo Fisher Scientific). After washing with PBS, cells were imaged and analyzed using a Thermo Scientific CellInsight CX5 High-Content Screening (HCS) platform. Infected cells were scored by perinuclear fluorescence above a set threshold determined by positive (untreated) and negative (uninfected) controls. A minimum of nine fields and 5,000 nuclei per well in triplicate or quadruplicate wells per treatment were scored in each experiment. All experiments were performed 2–4 times.

### SARS-CoV-2 Plaque Formation Assay (Italy/UniSR1/2020 Strain)

Experiments using live SARS-CoV-2 experiments were all performed in approved BSL 3/CL3 facilities by staff trained to work with BSL3/CL3 infectious organisms using standardized safety and decontamination protocols. Vero cells were plated at 2.5 × 10^5^ cell/well in 24-well plates in Essential-Modified Eagle Medium (EMEM, Lonza) supplemented with 10% FCS (EuroClone) (complete medium). Twenty-four hours later, cells were incubated with compounds in 250 μL of complete medium 1 h prior to infection and then incubated with virus suspension (pretreatment) containing 50 plaque-forming units (PFU) of Italy/UniSR1/2020 strain (GISAID accession ID: EPI_ISL_413489). After incubation for 1 h at 37°C, supernatants were discarded, and 500 µL of 1% methylcellulose (Sigma Chemical Corp) overlay dissolved in a complete medium was added to each well. Alternatively, Vero cells were incubated with compounds together with a virus suspension containing 50 PFU (cotreatment) in a total volume of 300 μL complete medium for 1 h. Supernatants were discarded and the methylcellulose overlay was added as described above. After 3 days, cells were fixed using 6% formaldehyde/PBS solution for 10 min and stained with 1% crystal violet (Sigma Chemical Corp) in 70% methanol for 1 h. The plaques were counted under a stereoscopic microscope (SMZ-1500, Nikon) and photographed using the EVOS M5000 system (Thermo Fisher).

### Quantitative Real-Time PCR for SARS-CoV-2

Cell culture supernatant from infection experiments was heat-inactivated at 56°C for 60 min following PHE protocols in the NHS Turnkey Labs based in the University of Birmingham Medical School. Viral RNA was reverse transcribed and quantified in the culture supernatant using the 1-step SARS-CoV-2 VIASURE Real-Time PCR Detection Kit (Prolab Diagnostics/CerTest Biotec) according to the manufacturer’s instructions. Briefly, 15 μL of rehydrated Reaction-Mix was combined with 5 μL of either heat-inactivated cell culture supernatant, positive virus RNA control, or negative control before cycling in an Agilent AriaMX Real-Time thermal cycler using the following cycle conditions: reverse transcription at 45°C for 15 min and initial denaturation at 95°C for 2 min followed by 45 cycles of 95°C for 10 s, 60°C for 50 s. Fluorimetric data were collected during the extension step for FAM (ORF1ab gene), ROX (N gene), Hex (internal control), and cycle thresholds (C_t_) calculated for each gene. Relative expression was calculated by subtracting the virus control Ct values from drug treatment samples and transforming the data using 2^−ΔCt^.

### Statistical Analysis

All pairwise comparisons were performed using paired *t*-tests or Mann–Whitney U tests where normal distribution was not assumed. Multiple comparisons were done using ANOVA.

## Results

### Validation of ACE2 Dimerization Assay

**FIGURE 1:**
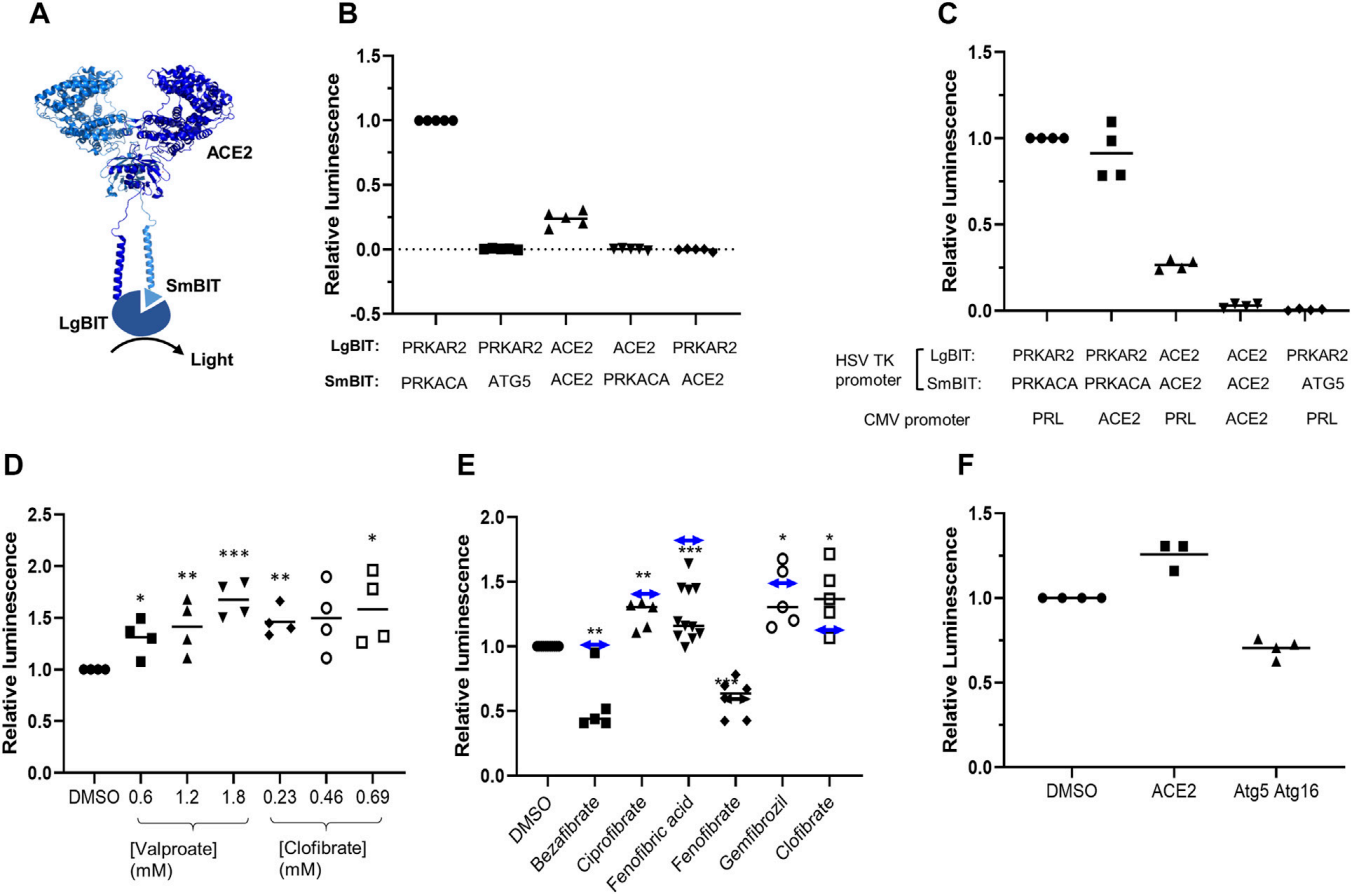
ACE2 dimerization assay. **A.** Schematic showing ACE2 tagged with LgBIT and SmBIT. **B.** HEK-293 cells were transfected with combinations of plasmids encoding LgBIT or SmBIT fused to either protein kinase A regulatory subunit (PRKAR2) or catalytic subunit (PRKACA), ATG5 or ACE2. The results (mean ± SD, n = 5) were normalized to the luminescence measured in cells transfected with protein kinase A reporters (positive control). **C.** HEK-293 cells were transfected with plasmids encoding ACE2 NanoBIT reporters under the control of the HSV TK promoter and ACE2 or prolactin (PRL) under the control of the CMV promoter. The results (mean ± SD, n = 4) were normalized to the luminescence measured in cells transfected with protein kinase A reporters and prolactin. **D.** HEK-293 cells were transfected with NanoBIT-tagged ACE2 reporters and incubated with sodium valproate or clofibrate at a concentration equal to 1x, 2x, or 3x the reported C_max_ of the drug. After 1 h, luminescence was measured and normalized (mean ± SD, n = 4) to that measured in cells treated with DMSO. **E.** A series of other fibrates were similarly evaluated in the assay. The luminescence measured (mean ± SD, n = 5–11) was significantly different to that measured in cells treated with solvent where shown (*p < 0.05; **p < 0.01; ***p < 0.005). When these fibrates were incubated with purified LgBIT and HiBIT-RBD to create a constitutively active NanoLuc, each of these fibrates was found to inhibit nanoluciferase (bezafibrate 35 ± 7%, ciprofibrate 55 ± 6%, fenofibric acid 48 ± 7%, fenofibrate 69 ± 5%, and gemfibrozil 61 ± 2% of the activity measured in the presence of DMSO). To correct this, the luminescence measurements from cells treated with fibrates in cells were divided by the fractional inhibition noted above to estimate the effect of the drugs on dimerization. These corrected results are shown as a horizontal line with double-headed arrows. **F.** HEK293 cells expressing either NanoBIT-tagged ACE2 or a combination of Atg5-SmBIT and Atg16-LgBIT were incubated with fenofibric acid (230 µM) and luminescence measured (mean ± SD, n = 4).

To develop an assay to measure dimerization of ACE2, two separate plasmids were created encoding ACE2 fused in frame at its C terminus to one of the NanoBIT reporters, SmBIT or LgBIT (Figure 1A). When these constructs were expressed in HEK293 cells, luminescence was observed that was approximately 20% of that generated by the protein kinase A positive controls comprised of LgBIT and SmBIT fused to the protein kinase A regulatory (PRKAR2) and catalytic (PRKACA) subunits, respectively (Figure 1B). Cotransfection of plasmids encoding ACE2 fused to either LgBIT or SmBIT and PRKAR2 or PRKACA subunits fused to the complementary NanoBIT reporter or co-transfection of NanoBIT-tagged ATG5 and PRKAR2, two proteins not known to interact, did not generate luminescence indicating that the assay measured ACE2 dimerization (Figure 1B). To further confirm the assay measured ACE2 dimerization, cells were transfected with a plasmid encoding untagged ACE2 as well as ACE2 tagged with LgBIT or SmBIT. The untagged ACE2 was expressed under the control of a CMV promoter, which provides substantially higher level expression than the HSV TK promoter which controls the expression of the NanoBIT-tagged ACE2. If the assay measures dimerization, the expression of the untagged ACE2 would be expected to suppress the luminescence by competing with the tagged ACE2 in dimers. To ensure that the effect observed did not result from competition for transcription factors, rather than as a result of the untagged ACE2 competing with NanoBIT-tagged ACE2, an unrelated gene (prolactin-PRL) was also expressed under the control of the CMV promoter. High-level CMV-driven expression of untagged ACE2, but not untagged PRL, suppressed the luminescence signal generated by ACE2-NanoBIT reporters (Figure 1C). Likewise, untagged ACE2 or PRL did not suppress the luminescence measured with the NanoBIT-tagged protein kinase A subunits (Figure 1C), thus confirming the specificity of the assay.

### Identification of ACE2 Dimerization Modulators

The ACE2-NanoBIT assay was used to screen a custom in-house library of approximately 100 approved drugs at a final concentration equal to their C_max_ in patients (FMC1 Library) (Khanim et al., 2011). Sodium valproate and clofibrate both increased the dimerization signal by approximately 33 and 56%, respectively. To confirm this, fresh compounds were purchased and retested at a concentration equal to their C_max_ in patients and multiples of this. Both compounds significantly increased the measured luminescence, confirming the results of the screen (Figure 1D). Although previously approved, clofibrate has subsequently been withdrawn due to unacceptable toxicity (Oliver, 2012). However, several other fibrates, bezafibrate, ciprofibrate, fenofibrate, and gemfibrozil, are still in clinical use. Apart from fenofibrate, these all bear a carboxylic acid, whereas fenofibrate is an isopropyl ester prodrug of fenofibric acid (Supplementary Figure S1). Noting that sodium valproate is also a lipophilic carboxylic acid, fenofibric acid was tested in the dimerization assay. All of the fibrates (tested at 230 μM, the C_ss_ of clofibrate (Männistö et al., 1975)) modestly, but significantly, increased luminescence (Figure 1E). However, they also substantially decreased the luminescence generated by mixing LgBIT with HiBIT-tagged RBD (which binds LgBIT with high affinity and independently of other interacting molecules). This suggested that the drugs inhibited NanoLuc directly and the measured luminescence underestimated dimerization. When the luminescence measured in the assay was corrected to take into account inhibition of nanoluciferase (Figure 1E), fenofibric acid emerged as the most effective, apparently increasing dimerization by approximately twofold. In contrast to this, fenofibrate did not increase dimerization. The increase in luminescence was also time-dependent, reaching a maximum after 30 min exposure to the drug (Supplementary Figure S2). To provide evidence for the specificity of the interaction, the effect of fenofibric acid on Atg5 and Atg16 tagged with LgBIT and SmBIT (Crowley et al., 2020) was investigated. Unlike ACE2, fenofibric acid did not increase the interaction of Atg5-Atg16 (Figure 1F).

To confirm these results, HEK-293 cells were transfected with plasmids encoding ACE2 tagged with streptavidin binding protein and a His-tag or ACE2 with a FLAG tag. Cells were exposed to drug and lysed and ACE2 complexes were purified using streptavidin beads. Following immunoblotting, ACE2-FLAG was only detected in lysates from cells transfected with both plasmids and not from cells transfected with one plasmid alone, confirming that the assay measured the interaction of ACE2. However, when cells were exposed to the fibrates, the amount of ACE2-FLAG detected on the beads was not substantially altered (Supplementary Figure S3).

### Effect of Fibrates on S Protein RBD

**FIGURE 2:**
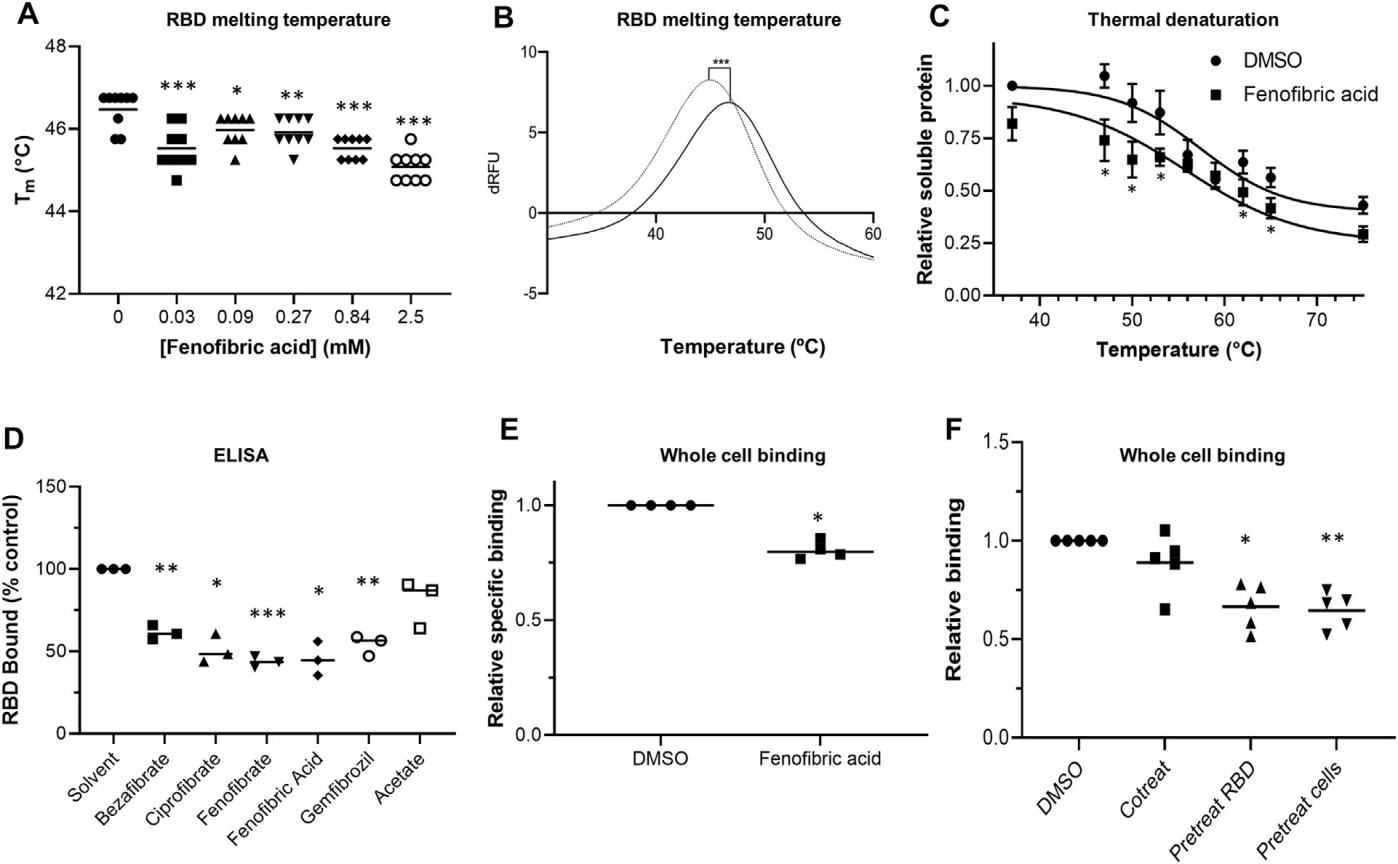
Effect of fenofibrate on RBD and RBD binding to ACE2 **A.** Differential scanning fluorimetry (DSF). T_m_ of 1 μg RBD alone or with increasing concentrations of fenofibric acid. The results (mean ± SD, n = 3) were significantly different from RBD where shown (***p < 0.001; **p < 0.01; *p < 0.05; paired *t*-test). **B.** The first differential of the thermal stability of 1 μg RBD alone (solid line) or with 2.5 mM fenofibric acid (dotted line). A direct interaction of fenofibric acid with SYPRO™ Orange dye (in the absence of RBD) was not observed. **C.** Cell culture supernatant containing HiBIT-RBD was incubated at the indicated temperature for 7 min and cleared by centrifugation, and the soluble RBD was measured by addition of HiBIT detection reagent. The samples also contained 0.05% DMSO or 230 µM fenofibric acid and are significantly different where shown (*p < 0.05). The results (mean ± SD, n = 4) are expressed as a fraction of the soluble RBD measured in the absence of drug. **D**. ELISA to measure inhibition of RBD binding to ACE2 by fibrates. Biotinylated ACE2 was captured onto a high binding microplate coated with streptavidin prior to the addition of RBD preincubated with or without 230 µM bezafibrate, ciprofibrate, fenofibrate, fenofibric acid, gemfibrozil, or acetate control. Data (mean ± SD, n = 3) represented % no inhibitor control and are significantly different from this where shown (*p < 0.05; **p < 0.01; ***p < 0.005). **E**. A whole cell-binding assay to measure inhibition of RBD binding to ACE2. COS cells were transfected with ACE2 and incubated on ice with HiBIT-tagged SARS-CoV-2 RBD and the indicated fibrate (230 µM). After washing, the bound RBD was measured by the addition of LgBIT and NanoLuc substrate. Nonspecific binding was measured in cells not expressing ACE2 and subtracted from the total binding to determine specific binding. The results mean ± SD, n = 4) were normalized to the binding measured in cells exposed to DMSO and are significantly different where shown (*p < 0.005). **F**. HiBIT-RBD or COS cells expressing ACE2 were preincubated (37°C) with fenofibric acid (230 µM) before mixing and measuring binding after a further 20 min. Nonspecific binding was measured as described above. The results (mean ± SD, n = 5) are significantly different (*p < 0.05; **p < 0.01) from the specific binding measured in cells in which HBIT-RBD and fenofibrate were added simultaneously to the cells.

To evaluate whether fibrates affect the viral spike protein RBD, the thermal stability of RBD in the presence and absence of fibrates was investigated using DSF. Changes in T_m_ of a protein in the presence of a ligand are indicative of binding and have previously been utilized to probe for protein-ligand interactions (Niesen et al., 2007). All of the fibrates altered T_m_ of RBD (46.4°C) although the greatest destabilization was observed with bezafibrate and ciprofibrate (ΔT_m_ = -1.9°C, (Supplementary Figure S4). A smaller effect was observed with fenofibric acid (ΔT_m_ = −1.4°C) but this was detectable at concentrations as low as 30 μM (Figures 2A,B). Although fenofibrate also destabilized RBD, this was only observed at higher drug concentrations (≥270 μM, Supplementary Figure S4). Acetate, a carboxylic acid lacking the lipophilic moieties found in the fibrates, had no significant effect on RBD T_m_ (Figure 2D, Supplementary Figure S4D) indicating that the lipophilic moieties are required. In addition, fenofibric acid also decreased the stability of the S1 protein (Supplementary Figure S4C).

To confirm these results, we used a modification of a cellular thermal shift assay in which aggregates of thermally unfolded protein are cleared by centrifugation. Consistent with the DSF assay, in this assay, fenofibric acid decreased T_m_ of RBD by 2-3°C (Figure 2C).

### Fenofibric Acid Inhibits ACE2-RBD Binding

An ELISA consisting of immobilized, recombinant ACE2 was employed to determine the inhibitory effect of fibrates on RBD-ACE2 binding. All fibrates screened demonstrated significant inhibition of binding at a concentration of 230 μM, the C_max_ of clofibrate (Figure 2D). The binding of RBD to ACE2 expressed in COS cells was measured as previously described (Lima et al., 2021). When these assays were conducted on ice to minimize endocytosis and a membrane impermeant detection reagent was used to detect extracellular binding, no inhibition of RBD binding was observed with any of the fibrates (Supplementary Figure S5A) and in some cases, there was apparently a modest stimulation. The assay was adapted for use at 37°C and reached a steady state by 20 min (data shown). Using this revised protocol, fenofibric acid was found to modestly, but significantly, inhibit RBD binding to ACE2 (Figure 2E). This was not due to toxicity as 99 ± 1% (n = 4) of the cells excluded trypan blue after similar exposure to drug. Furthermore, when either RBD or cells expressing ACE2 were preincubated with fenofibric acid, significantly more inhibition of the binding was observed than when all three were coincubated, consistent with the drug affecting both RBD and ACE2 (Figure 2F). Lastly, in a preliminary experiment, fenofibric acid inhibited binding to fixed Vero cells (Supplementary Figure S5). These data indicate that fenofibrate/fenofibric acid interferes with spike RBD binding to ACE2.

### Fenofibrate Inhibits Infection of Vero Cells by the hCOV-19/England/2/2020 Virus Isolate

**FIGURE 3:**
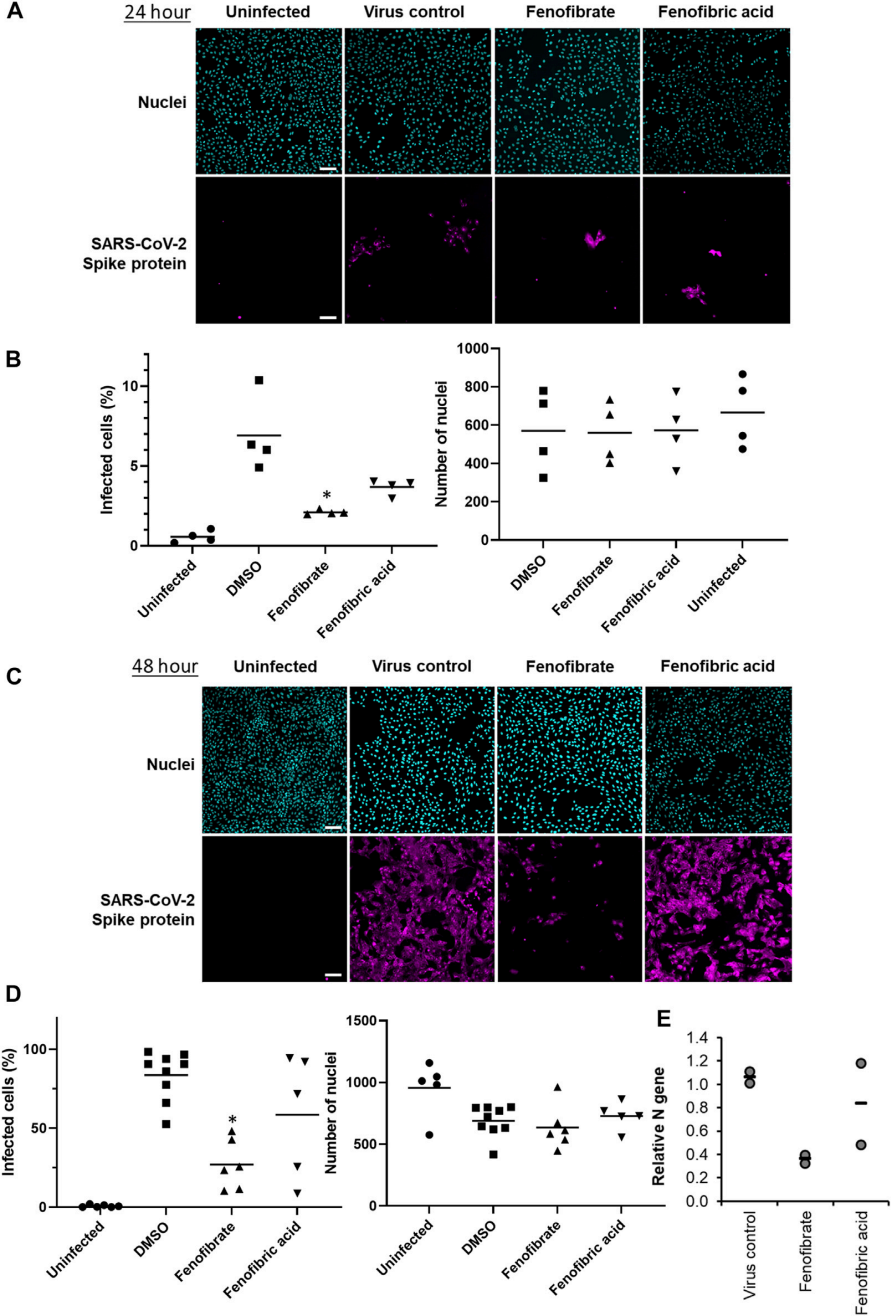
Fenofibrate and to a lesser extent fenofibric acid reduce SARS-CoV-2 infection at both 24 and 48 h. Vero cells were plated into 96-well plates (8 × 10^3^ cells/well) for 24 h before infecting with 167IU of hCOV-19/England/2/2020 virus isolate in the absence or presence of 230 μM fenofibrate or fenofibric acid. Infection rates were assessed at 24 and 48 h by staining Vero cells for viral spike protein and counterstaining nuclei with Hoechst. Cells were imaged and analyzed using a Thermo Scientific CellInsight CX5 HCS platform. Representative images and mean data are shown for Vero cells incubated for 24 h (**A** and **B**) and 48 h (**C** and **D**). The black bars are % infected cells and the hatched gray bars are the average number of nuclei scores per field of view (mean ± SD, n = 2-3; one-way ANOVA, *p < 0.05 compared to virus control). **E.** Supernatant was collected from wells after 48 h of incubation. Virus was heat-inactivated and viral N gene RNA levels were measured directly in the supernatant using a commercial one-step RT-qPCR reaction. N RNA levels were calculated relative to supernatant from virus control (n = 4 experiments).

To evaluate the potential therapeutic effect of fenofibrate/fenofibric acid on SARS-CoV-2 virus, infection experiments were performed independently in two separate laboratories. Using the hCOV-19/England/2/2020 virus strain, Vero cells were coincubated with virus and fibrates before fixing and staining for spike protein and counterstaining nuclei with Hoechst. Analysis after 24 h incubation of Vero cells with SARS-CoV-2 virus identifies changes in primary infection rates, whereas by 48 h, virus particles released by infected Vero cells into the culture medium infect other cells in the wells. Thus, analysis at 48 h can also identify potential actions of drugs on viral replication, release, and infection. By 48 h, 59% of Vero cells stained positive for spike protein in virus control wells with minimal loss of cell numbers (Supplementary Figure S6). Consistent with the binding assays and of the fibrates studied (all screened at 230 µM), only fenofibrate reduced virus infection by ∼65–18% compared to virus control (Supplementary Figure S6). This was not attributable to loss of Vero cell viability as no decrease in cell number by fenofibrate was seen as measured by the number of nuclei and by Cell Titer Blue assay (Supplementary Figure S8). No difference was observed when cells were pretreated or cotreated with drug and virus (data not shown). Parallel experiments were performed with a panel of statins (simvastatin, pitavastatin, rosuvastatin, and pravastatin, (Supplementary Figure S1), drugs which have largely replaced fibrates as front-line therapy for treating lipid disorders. When screened at 100 nM, neither pravastatin nor rosuvastatin reduced infection and had no effect on Vero cell viability as measured by the number of nuclei (Supplementary Figures S6–8). At this concentration both simvastatin and pitavastatin were apparently cytotoxic. Titration experiments (Supplementary Figure S8) indicated that, at 10 nM, simvastatin and pitavastatin did not affect cell number but when tested at this concentration, like the hydrophilic statins, these lipophilic statins also did not reduce viral infection (Supplementary Figures S6,7). Subsequent experiments assessed the effect of fenofibrate and fenofibric acid on infection by SARS-CoV-2. Within 24 h, fenofibrate had significantly reduced infection levels by ∼60% indicating that fenofibrate is able to inhibit primary infection (Figure 3A,B). A reduction was also observed with fenofibric acid, albeit less than fenofibrate; however, the results were more variable in the experiments performed and did not reach significance (Figures 3A,B). This pattern was recapitulated at 48 h (Figures 3C,D) indicating that suppression of infection by fenofibrate is sustained. These data indicate that, in this setting, fenofibrate and, to a lesser extent, fenofibric acid are able to reduce primary infection and also secondary infection rates.

**FIGURE 4:**
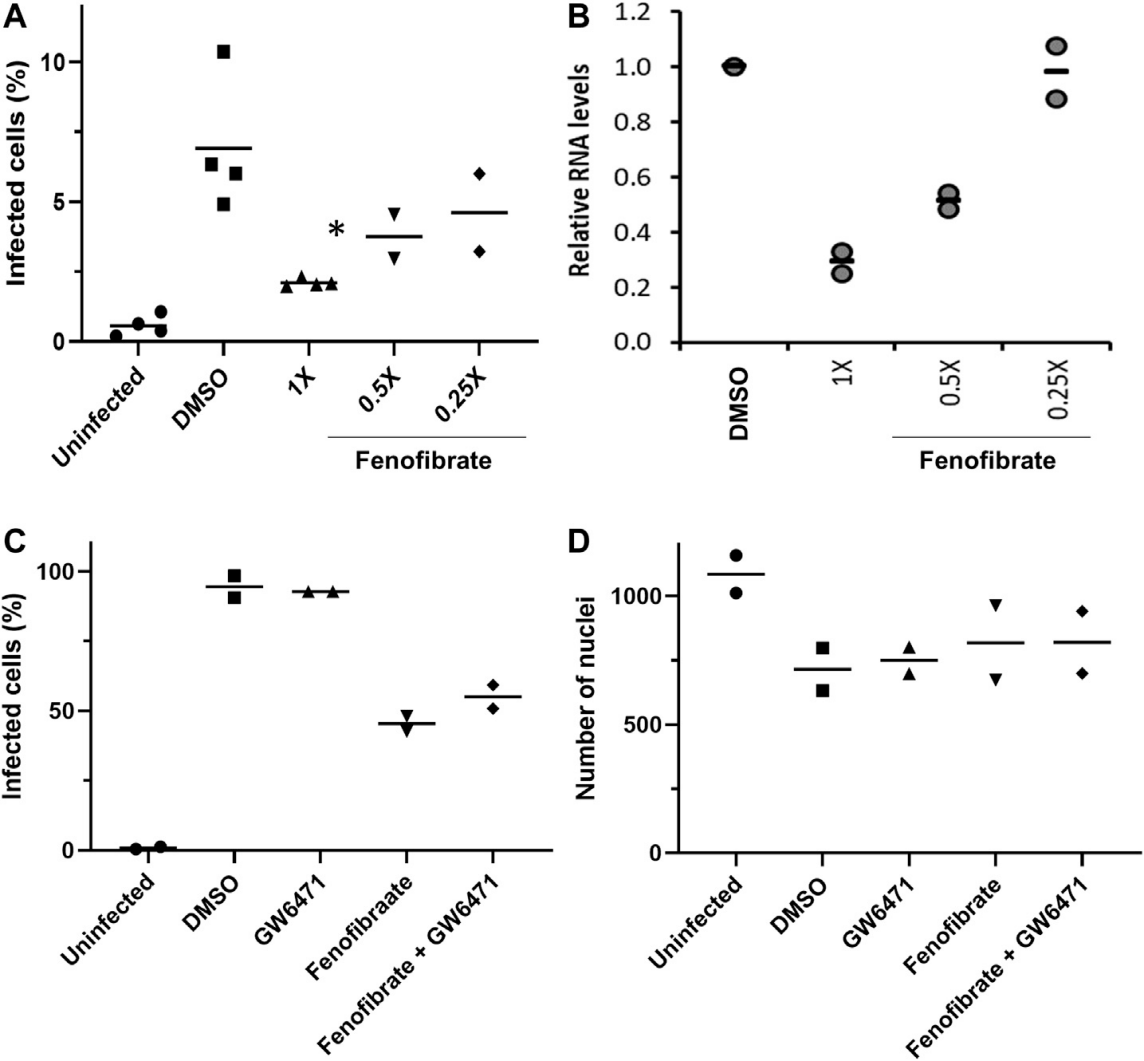
Fenofibrate reduces SARS-CoV-2 infection level in vitro in a dose-dependent manner. Vero cells were plated into 96-well plates (8 × 10^3^ cells/well) for 24 h before infecting with 167 IU of hCOV-19/England/2/2020 virus isolate in the absence or presence of 1x (230 μM), 0.5x, or 0.25x fenofibrate. Infection was assessed at 24 h by staining Vero cells for viral spike protein and counterstaining nuclei with Hoechst. Cells were imaged and analyzed using a Thermo Scientific CellInsight CX5 HCS platform. **A**. Mean infection rates observed at 24 h (n = 2–3). **B.** Supernatant was collected from wells after 48 h of incubation. Virus was heat-inactivated and viral N gene RNA levels were measured directly in the supernatant using a commercial one-step RT-qPCR reaction. N RNA levels were calculated relative to supernatant from virus control (n = 4). To determine the role of PPARα, 48 h infection experiments were performed in the absence or presence of the PPAR-alpha antagonist GW6471 (1 μM). Mean data from two to three experiments are shown in (**C** and **D**). **C** shows % infected cells and **D** the average number of nuclei scores per field of view. Statistical significance was calculated by one-way ANOVA. *p < 0.05 compared to virus control.

To determine virus levels in cell culture supernatant, virus RNA levels were measured by multiplex qRT-PCR for viral ORF1ab and N genes on heat-inactivated culture supernatant from 48 h experiments. While ORF1ab RNA levels were detectable in virus control supernatant, no signal was detected in supernatant from drug-treated cells implying a reduction in virus RNA (data not shown). However, a signal for the viral N gene was detectable by qRT-RCR in all samples. Consistent with the reductions seen in infection levels, fenofibrate significantly reduced viral N gene RNA levels, whereas the results with fenofibric acid were more variable (Figure 3E). Furthermore, the effect of fenofibrate on infection rates and viral RNA levels in culture supernatant was dose-dependent as determined by doubling dilution experiments (1x: 230 μM; Figures 4A,B). Fenofibrate works as an antihyperlipidaemia agent by acting as a PPARα agonist. Treatment with the PPARαantagonist GW6471 did not appreciably alter the antiviral actions of fenofibrate (Figures 4C,D) in this setting.

### Fenofibrate Inhibits Infection of Vero Cells by the Italy/UniSR1/2020 Virus Isolate

**FIGURE 5:**
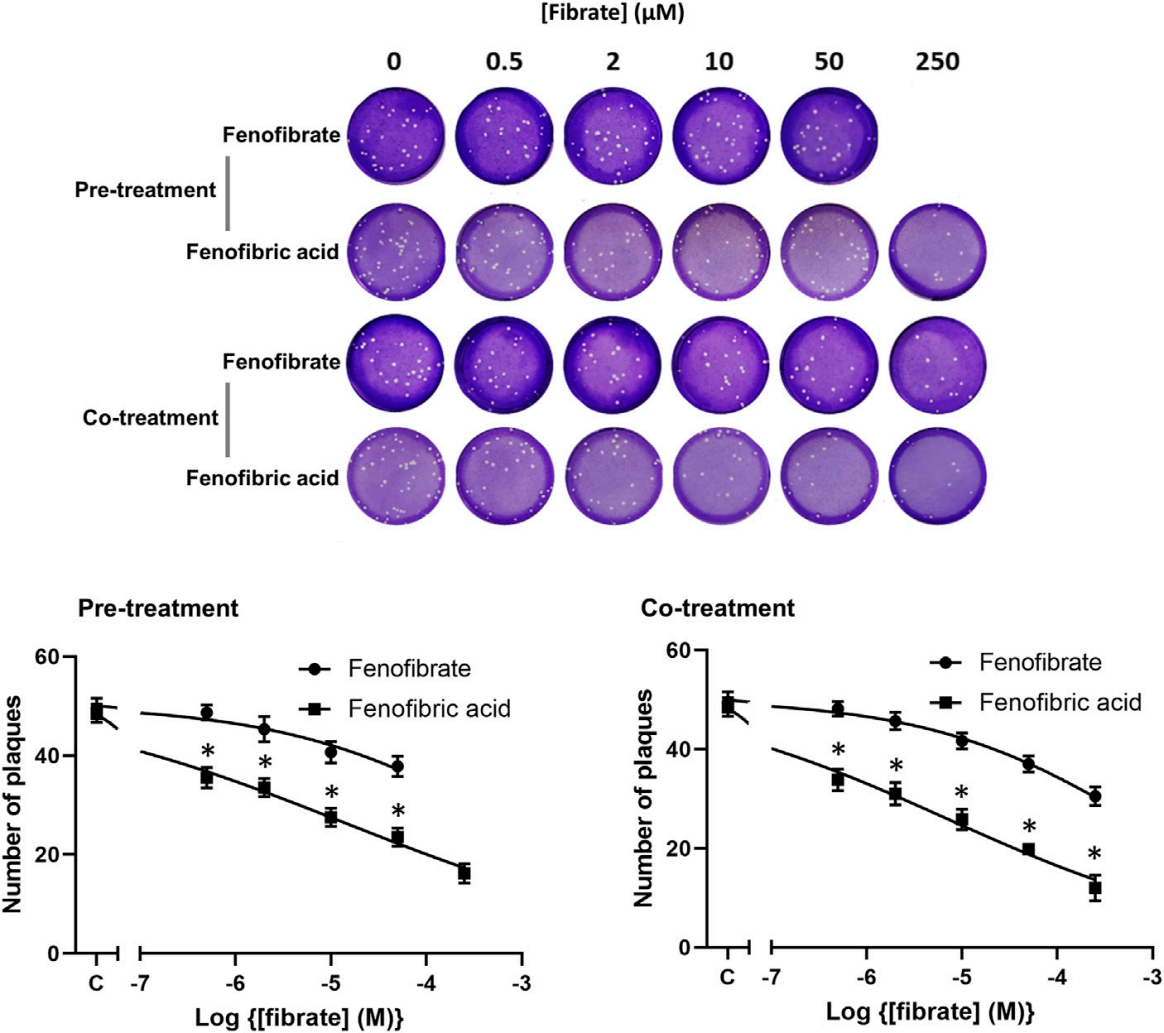
Fibrate inhibition of SARS-COV-2 infection of Vero cells. Antiviral effect of fibrates added 1 h before infection or in cotreatment with infection in Vero cells with 50 PFU of SARS-CoV-2. ND, not determined due to solubility issues. The results are expressed as the number of PFU/well (mean ± SD, n = 3). The number of plaques was significantly different (2-way ANOVA) in cells treated with fenofibric compared to fenofibrate where shown DMSO (*p < 0.001). Compared to cells treated with drug solvent, the number of plaques was significantly different in cells treated with fenofibric acid (*p* < 0.001, all concentrations tested) and in cells treated with fenofibrate (*p* < 0.01, 10 µM fenofibrate; *p* < 0.001, 50 µM fenofibrate).

To confirm the infection results observed with hCOV-19/England/2/2020 isolate in experiments performed at the University of Birmingham, the effect of fenofibrate and fenofibric acid was assessed on plaque formation in Vero cells infected with the Italy/UniSR1/2020 SARS-CoV-2 isolate independently at San Raffaele Scientific Institute in Milan. Both the hCOV-19/England/2/2020 isolate and Italy/UniSR1/2020 isolate are identical to the original Wuhan viral strain. Vero cells were pretreated for 1 h with fenofibrate or fenofibric acid (“pretreatment”). Alternatively, the cells were exposed to the drug and the virus at the same time (cotreatment). After 1 h, the virus was removed and plaques were allowed to form. Fenofibric acid inhibited plaque formation at concentrations clinically achievable in patients (pretreatment IC_50_ 14 μM; cotreatment IC_50_ 7 μM) (Figure 5). Fenofibrate also reduced the number of plaques formed, but notably less potently. As observed for the hCOV-19/England/2/2020 strain, there was no substantial difference between the pretreatment and cotreatment experiments.

Thus, using two different virus isolates, we demonstrate that fenofibrate or its active metabolite fenofibric acid are able to significantly reduce SARS-CoV-2 infection in cell culture models.

## Discussion

The development of new more infectious SARS-CoV-2 variants has resulted in a rapid expansion in infection rates and deaths in several countries around the world, especially the United Kingdom, US, and Europe. While vaccine programs will hopefully reduce infection rates and virus spread in the longer term, there is still an urgent need to expand our arsenal of drugs to treat SARS-CoV-2-positive patients. Using an unsupervised approach, we have identified that the off-patent licensed drug fenofibrate has the potential to treat SARS-CoV-2 infections. The drug was identified through a screen of approved drugs to identify those which alter the dimerization of ACE2. Clofibrate was identified as a hit in this screen and testing of other fibrates led to the identification of fenofibrate as being the most likely to be effective as an antiviral agent. Fenofibric acid also appears to affect the stability of spike protein RBD and inhibit binding to ACE2. Importantly, these effects on RBD by fenofibric acid/fenofibrate correlated with decreases in SARS-CoV-2 infection rates *in vitro* using two different virus assays (staining for spike protein and plaque formation) in two independent laboratories.

**FIGURE 6:**
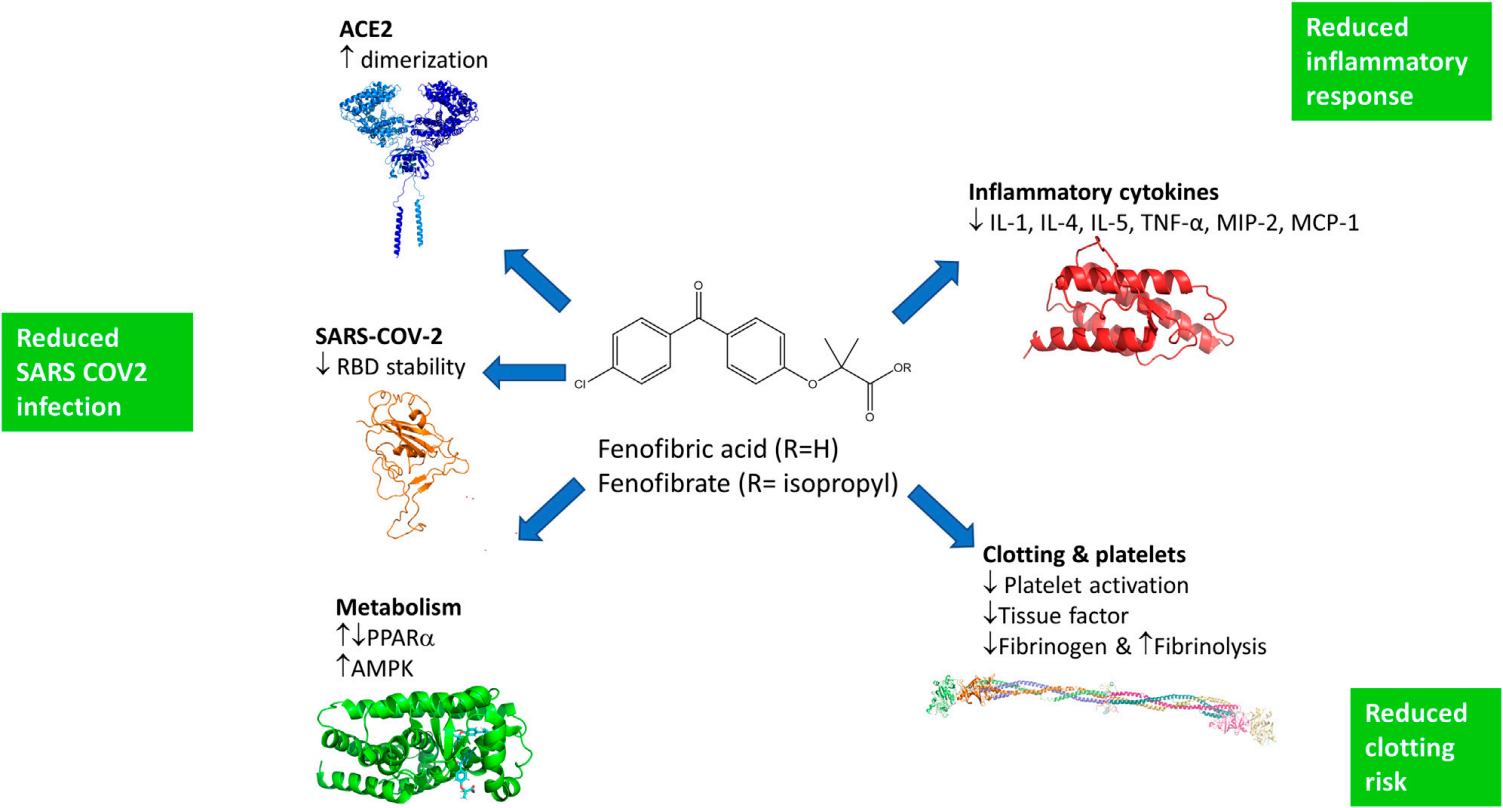
Potential mechanisms by which fenofibrate may improve the treatment of SARS-COV-2 infections. Fenofibric acid, the metabolite of fenofibrate, stimulates ACE2 dimerization, destabilizes the RBD, and exerts metabolic effects which are likely to reduce infection. Fenofibric acid possesses anti-inflammatory properties which are likely to blunt the immune response and correspondingly alleviate symptoms. Lastly, fenofibric acid inhibits platelet activation and aggregation, which is anticipated to reduce the hemodynamic problems seen in SARS-COV2 patients.

The ACE2 dimerization assays depend on the colocalization of LgBIT and SmBIT brought about by the formation of ACE2 dimers. No signal was observed using protein kinase A subunits that do not interact with ACE2 and overexpression of unlabeled ACE2 suppressed the signal from the NanoBIT reporters, giving confidence that the assay measures the interaction of ACE2 protomers. Although described here as a dimerization assay, the assay may not discriminate between dimer formation and higher-order oligomers, and drugs showing activity in the dimerization assay could alternatively elicit conformational changes in ACE2 complexes which improve the interaction of the NanoBIT reporters. We also acknowledge that although ACE2 is well-established as a membrane protein and this is supported by our own binding assays, we have not formally shown that the NanoBIT-tagged proteins are located on the cell membrane. All the fibrates tested showed some activity in the dimerization assays, but the most pronounced effects were observed with fenofibric acid. Following oral administration of fenofibrate, the ester prodrug is completely converted to the free acid (Figure 6) in a reaction thought to be catalyzed by carboxylesterases. The prodrug fenofibrate (the isopropyl ester of fenofibric acid) was inactive in the dimerization assay, suggesting that the free carboxylic acid is necessary.

In addition to effects on ACE2, DSF showed that all the fibrates destabilized the viral spike protein RBD and lowered its “melting” temperature. The most potent effects were again seen with fenofibric acid. These results were corroborated with a modified “CETSA” assay which measured RBD aggregation after thermal denaturation. The effects of fenofibric acid on RBD may contribute to its inhibition of the binding of RBD to ACE2 in ELISA and cell-binding studies performed at 37°C. When measured in cells at 0°C, the fibrates did not inhibit binding to ACE2; this temperature is likely to prevent melting, providing a potential explanation for the lack of activity of fibrates in binding assays at lower temperatures. To provide evidence for a dual mechanism of action of fenofibrate on both RBD and ACE2, we compared preincubation of cells or RBD with fenofibric acid to experiments when binding of RBD was measured when all three reagents were coincubated. Preincubation of fenofibrate with either ACE2-expressing cells or RBD increased the inhibition of RBD binding by fenofibric acid, consistent with the drug having effects on both RBD and ACE2. Taken together, these data prompted us to evaluate whether fenofibric acid or fenofibrate would reduce infection by SARS-CoV-2.

To provide robust data evaluating the potential of fenofibric acid/fenofibrate to inhibit infection by SARS-CoV-2, the drugs were evaluated independently in two separate laboratories using different viral infection assays performed on Vero cells and two separate SARS-CoV-2 isolates, both of which are identical to the original Wuhan strain (hCOV-19/England/2/2020 and Italy/UniSR1/2020). In both cases, fenofibrate/fenofibric acid was found to significantly reduce infection rates. Fenofibrate/fenofibric acid decreased the number of Vero cells staining positive for viral spike protein at 24 h indicating inhibition of primary infection. The number of cells infected 48 h after infection was also significantly reduced, demonstrating the potential for sustained inhibition of infection. This was further confirmed by PCR which showed a reduction in viral mRNA released by the cells into the culture supernatant. Likewise, we saw significant reductions with fenofibric acid/fenofibrate in plaque formation assays which are considered the gold-standard assay for measuring infectivity by SARS-CoV-2. Several assays demonstrate that the reduced viral infection was not due to a cytotoxic effect of the fibrates in the host cells. Considering that fenofibrate is used in the treatment of hypercholesterolaemia and hyperlipidaemia, the effect of several statins on SARS-CoV-2 infection was also assessed. These included both hydrophilic (pravastatin; rosuvastatin) and lipophilic statins (pitavastatin; simvastatin). None of the statins inhibited viral infection, suggesting that the antiviral effect was not mediated by inhibition of cholesterol synthesis. The differences we observed in potency between fenofibrate and fenofibric acid in the two antiviral assays may reflect different strains of the virus or different methodologies. Although we cannot presently fully explain these, it is clear that fenofibrate or its metabolite fenofibric acid demonstrated anti-SARS-CoV-2 activity.

Fenofibric acid was identified as a potential antiviral agent through its effects on ACE2 dimerization, but it remains to be clarified to what extent the effects of fenofibrate/fenofibric acid on dimerization contribute to its antiviral activity. The mechanism by which increased dimerization could inhibit viral infection was not investigated and several explanations are plausible. It was not possible to measure the effect of fibrates on dimerization of ACE2 in streptavidin precipitation assays. This may reflect the insensitivity of this latter method or that fenofibrate alters the conformation of ACE2 rather than inducing dimerization. Structural studies have shown that ACE2 adopts “open” and “closed” conformations (Yan et al., 2020) which may be detected by the NanoBIT reporters. The open and closed conformations may also affect RBD binding to each ACE2 protomer or the number of spike proteins that can bind to an ACE2 dimer, thereby affecting the avidity of the virus for cells. Conformational changes in ACE2 may also affect its susceptibility to proteolysis by TMPRSS2. The suggestion that the antiviral activity of fenofibrate depends at least in part on effects on ACE2 also offers advantages over drugs that inhibit viral proteins. Mutations in the viral genome are less likely to affect the antiviral activity of drugs which target human rather than viral proteins. Excitingly, fenofibrate also destabilized the RBD and reduced its binding to ACE2. It is highly likely that this contributes to the reduced infection in cells treated with fenofibrate. This also suggests that fenofibrate has multiple mechanisms of action, making it less likely that resistance to it will quickly emerge and fenofibrate may retain activity against newly emerging strains of SARS-CoV-2. However, our data suggest that the antiviral activity of fenofibrate measured in the infection assays presented here is not mediated by the transcription factor PPARα. The efficacy of fibrates in the treatment of hyperlipidaemia depends on their ability to activate PPARα However, GW6471, a PPARα antagonist (Xu et al., 2002), did not prevent fenofibrate from inhibiting viral infection.

To our knowledge, this is the first experimental evidence that fenofibrate can modulate RBD and ACE2 proteins and inhibit SARS-CoV-2 infection. Importantly, others have also proposed its therapeutic use in SARS-CoV-2. These proposals are based on pharmacological effects of fenofibrate that are additional to the ones we have identified here (summarized in Figure 6). Fenofibrate increases the levels of the glycosphingolipid sulfatide and this has been proposed to reduce SARS-CoV-2 infection (Buschard, 2020). SARS-CoV-2 infection is associated with overproduction of cytokines, such as TNF-α, IFN-γ, IL-1, IL-2, and IL-6, and subsequently a cytokine storm that induces several extrapulmonary complications including myocardial injury, myocarditis, acute kidney injury, impaired ion transport, acute liver injury, and gastrointestinal manifestations such as diarrhea and vomiting (Gupta et al., 2020; Lee and Choi, 2021). Similar to dexamethasone, fenofibrate has been shown to suppress airway inflammation and cytokine release including TNF-α, IL-1, and IFN-γ in both mouse and human studies (Madej et al., 1998; Delayre-Orthez et al., 2008; Stolarz et al., 2015). Fenofibrate has also been shown to have antithrombotic and antiplatelet activities (Jeanpierre et al., 2009; Lee et al., 2009) reduce fibrinogen levels and increase clot permeability, thereby enhancing fibrinolysis (Undas et al., 2006). These properties may reduce or prevent hypercoagulability seen in the late stage of disease in many SARS-CoV-2 patients (Rogosnitzky et al., 2020). A meta-analysis has also suggested that fenofibrate may be useful in the treatment of hepatitis C infection (Grammatikos et al., 2014). Lastly, we note a preprint from the group of Nahmias that has also suggested that fenofibrate may have clinical effects against SARS-CoV-2 infection which depends on the PPARα mediated alterations in host cell metabolism (Ehrlich et al., 2020). Based on the data in this preprint, two clinical trials have been registered using fenofibrate in SARS-CoV-2 patients requiring hospitalization (Hospital of the University of Pennsylvania (NCT04517396) and Hebrew University of Jerusalem (NCT04661930)). The metabolic effects of fenofibrate may be mediated not only by its cognate target, PPARα, but also by activation of AMPK (Murakami et al., 2006) which regulates protein synthesis and autophagy pathways through mTORC1.

Given the current acceleration in infection and death rates observed in several countries, we strongly advocate clinical trials of fenofibrate in patients with SARS-CoV-2 requiring hospitalization. Fenofibrate has a relatively safe history of use, the most common adverse effects being abdominal pain, diarrhea, flatulence, nausea, and vomiting. The half-life of fenofibric acid is 20 h (Desager et al., 1996), allowing convenient once daily dosing. The recommended doses in the United Kingdom (up to 267 mg) provide plasma concentrations (Cmax 70 μM; Css 50 µM) comparable to those at which we and others have seen antiviral activity, Finally, if proven effective, fenofibrate is available as a “generic” drug and consequently is relatively cheap, making it accessible for use in all clinical settings, especially those in low and middle-income countries. Preliminary data indicate that fenofibrate is equally effective against the B.1.1.7 variant (data not shown) implying that mutations in S protein are unlikely to affect the efficacy of fenofibrate. There are a number of medical conditions which contraindicate the use of fenofibrate, such as significantly impaired kidney function, and these could potentially limit its use in the treatment of COVID patients. There are also a number of drug interactions with fenofibrate which are potentially severe, although some of these may be avoided by temporarily withholding the interacting drug. Appropriate risk-benefit analysis will be necessary once the clinical antiviral activity of fenofibrate is defined to identify which SRS-COV2 patients can safely be treated with fenofibrate. While further studies to clarify the precise mechanism of the antiviral activity of fenofibrate are ongoing, our data support the clinical evaluation of fenofibrate in the community infection setting and also in patients requiring hospitalization. One possibility is that fenofibrate is tested in newly diagnosed symptomatic patients, who do not require hospitalization, in whom reduction in viral infection levels by fenofibrate would reduce disease severity and the spread of infection to other individuals.

## Data Availability

The raw data supporting the conclusions of this article will be made available by the authors, without undue reservation, to any qualified researcher.

## Abbreviations

Css, steady-state plasma concentration; Cmax, maximum plasma concentration; LgBIT: large binary interaction technology; HiBIT: high affinity binary interaction technology; RBD: receptor-binding domain; ACE2: angiotensin-converting enzyme 2; SARS: severe acute respiratory syndrome; ELISA: enzyme-linked immunosorbent assay
