# Supplementary material for: The Hyperlipidaemic Drug Fenofibrate Significantly Reduces Infection by SARS-CoV-2 in Cell Culture Models
Source: Front Pharmacol. 2021 Aug 6;12:660490. doi: 10.3389/fphar.2021.660490 (PMC8377159; doi:10.3389/fphar.2021.660490)
Supplement: Supplementary file 1 [file DataSheet1.PDF]

Supplementary material

The hyperlipidaemic drug fenofibrate significantly reduces infection by SARS-CoV-2 in cell culture models

Scott P. Davies, Courtney J. Mycroft-West, Isabel Pagani, Harriet J. Hill, Yen-Hsi Chen, Richard Karlsson, Ieva Bagdonaite, Scott E. Guimond, Zania Stamataki, Marcelo Andrade De Lima, Jeremy E. Turnbull, Zhang Yang, Elisa Vicenzi, Mark A. Skidmore, Farhat Khanim* and Alan Richardson*

*Corresponding authors :

a.richardson1@keele.ac.uk ,

F.L.Khanim@bham.ac.uk

This PDF file includes: Figs. S1 to S8

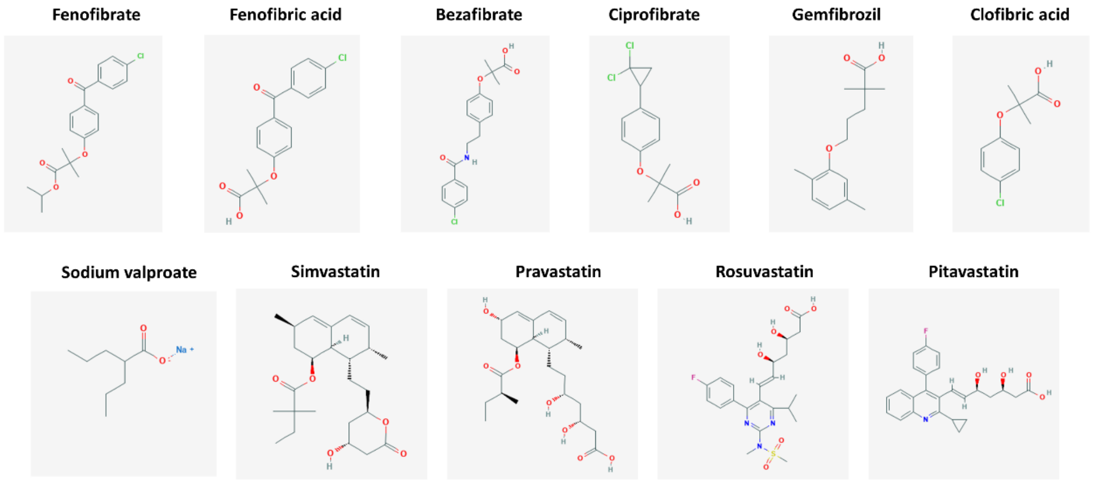

Figure S1 Chemical structure of drugs evaluated. Structures were downloaded from pubchem.

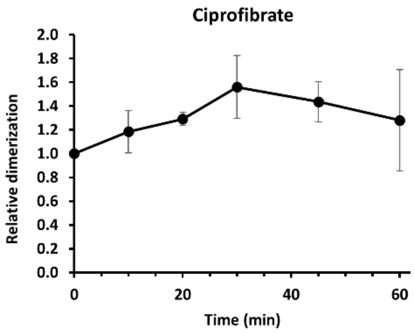

Figure S2 Kinetics of ciprofibrate induced ACE2 dimerization. HEK-293 cells were transfected with plasmids encoding ACE2 nanoBIT reporters. After 48 hours, the cells were treated with 230 µM ciprofibrate and the luminescence measured. The results are expressed as a proportion of that measured in cells treated with solvent (mean ± S.D>, n =3).

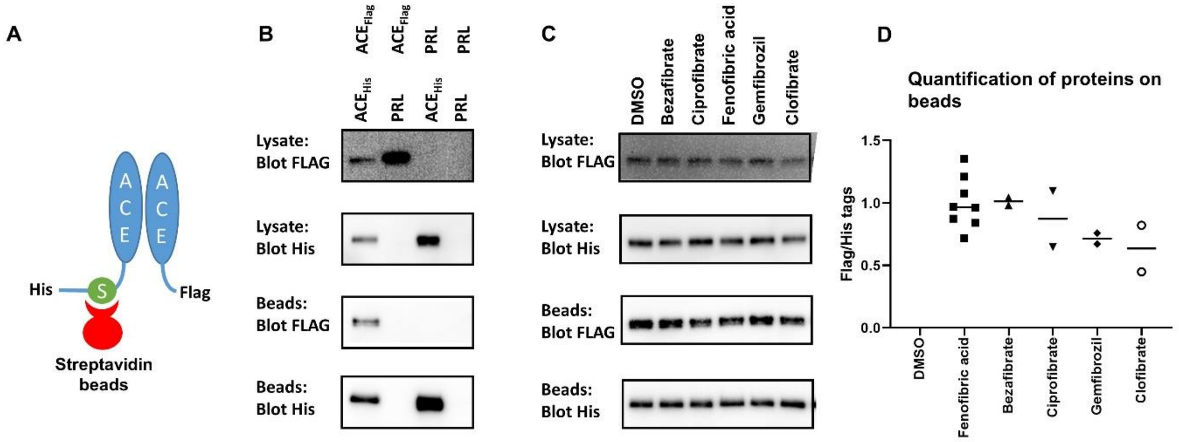

Figure S3. Measurement of ACE2 dimers by collection on streptavidin beads. A. Schematic showing the principle of the assay. ACE2 complexes were measured in HEK-293 cells expressing ACE2 fused to Streptavidin binding protein (S) and a His tag or encoding ACE2 with a flag tag. **B** Cells were transfected with plasmids encoding the His tagged protein (ACE_{His}) or the Flag-tagged protein (ACE_{Flag}) or prolactin (PRL) if one of the constructs encoding aa tagged ACE was omitted. ACE2 complexes were purified fusing streptavidin beads and analysed by immunoblotting. The results are representative of three experiments. **C**. HEK-293 cells expressing the tagged ACE2 proteins were exposed to the indicated drugs (230 µM) for 1 hour and the ACE2 complexes purified and analysed as described in B. The results are representative of three experiments. **D.** The His and Flag blots were quantified (Alpha view software), normalized to DMSO controls and then expressed as ratio of Flag/His to assess the effect of the drugs on dimerization. All data points are shown.

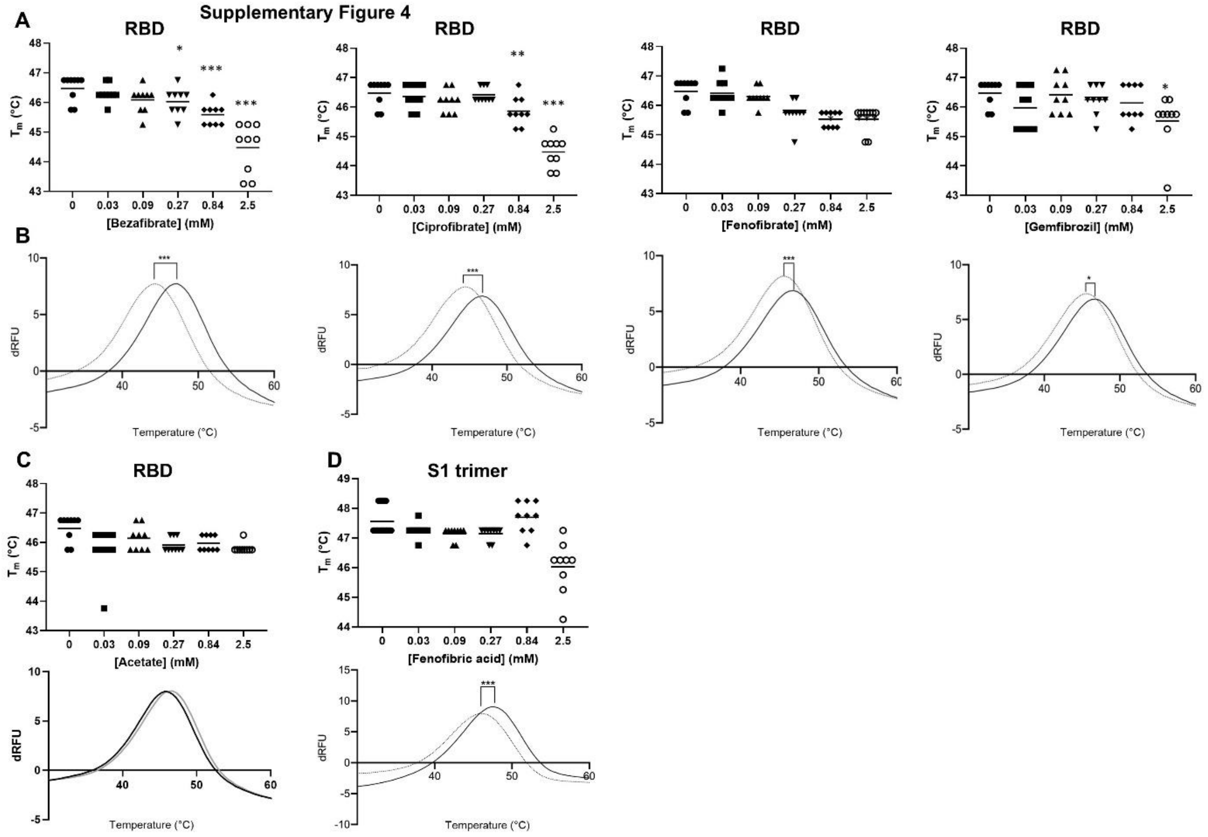

Figure S4. Differential scanning fluorimetry. A. The T_m of 1 µg RBD alone or with increasing concentrations of the indicated fibrate. The results (mean ± S.D., n=3) were significantly different from RBD where shown (***, $P<0.001$; **, $P<0.01$; *, $P<0.05$; paired t-test). **B.** First differential of the thermal stability of 1 µg RBD alone (solid line) or with 2.5 mM fibrate (dotted line). Fenofibrate displayed a direct interaction with SYPRO™ Orange dye at the highest concentration tested (2.5 mM); as a consequence, this data point has been omitted. No interaction was observed between fenofibrate and SYPRO™ Orange at concentrations ≤ 0.84 mM, at which concentration a significant difference can be overserved between the T_m of RBD alone and that in the presence of 0.84 mM fenofibrate, displaying a ΔT_m of 0.9 °C. All fibrates screened induced a comparable shift in T_m at 0.84 mM, with the exception of gemfibrozil, which did not display a significant change in T_m. **C.** The same assay was conducted with acetate (mean ± S.D., n=3) as a negative control containing a carboxylic acid but lacking a significant lipophilic moiety. **D.** The same assay was conducted with fenofibric acid, measuring binding to S1 trimer.

Supplementary Figure 5

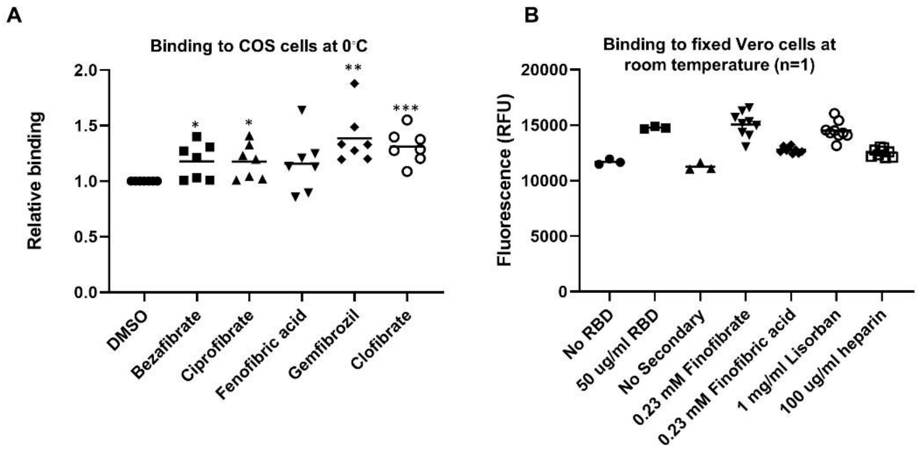

Figure S5 Binding of RBD to ACE2. A. Binding to live COS cells at 0°C. COS cells were transfected with ACE2 and binding of RBD-HiBIT, separately expressed in COS cells, measured as described(12). The results (mean ± S.D, n=7) are expressed as proportion of the binding to cells treated with solvent alone and are different to it where shown (*, P<0.05; **, P<0.01, ***. P<0.005). **B.** Binding or RBD to ACE2 in formalin fixed Vero cells was measured in the presence of the indicated drug. This is a preliminary experiment performed once with each sample being have 3-9 replicates.

Figure S6

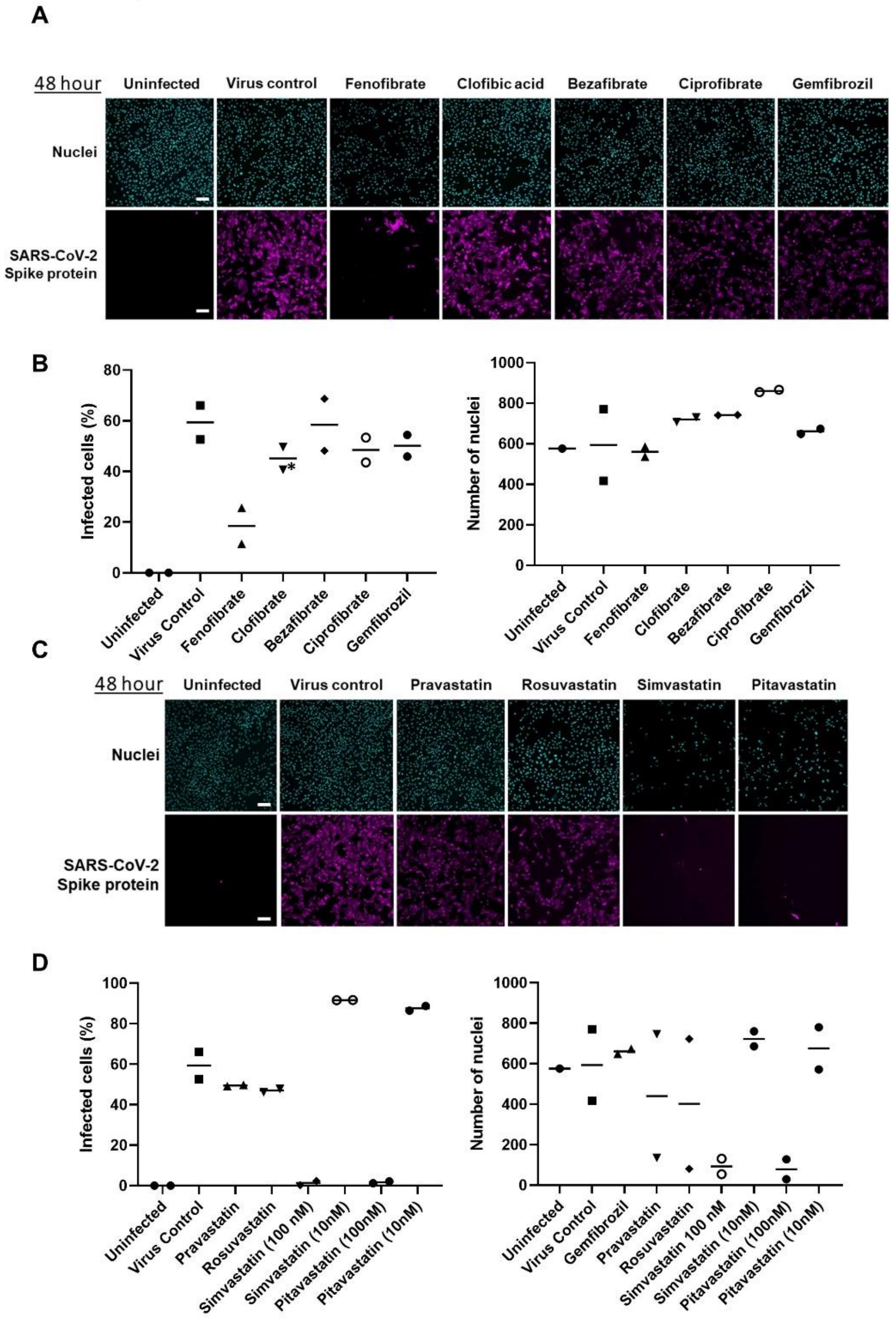

Figure S6. Fenofibrate reduces SARS-CoV-2 infection rates in vitro. Vero cells were plated into 96 well plates (8 x 10^3 cells/well) for 24 hours before infecting with 167 IU of hCOV-19/England/2/2020 virus isolate in the absence or presence of drugs. Infection rates were assessed at 48 hours by staining Vero cells for viral Spike protein and counterstaining nuclei with Hoescht. Cells were imaged and analysed using a Thermo Scientific CellInsight CX5 High-Content Screening (HCS) platform. Representative images and mean data are shown for Vero cells incubated with either no virus, SARS-CoV-2 virus control, or virus and fibrates (230μM, (**A and B**) or statins (100nM, **C and D**). The black bars are % infected cells and the hatched grey bars are average number of nuclei score per field of view (mean ± S.D., n=2-3; one-way ANOVA. *, $P < 0.05$ compared to virus control).

Figure S7. Statins do not reduce infection of Vero cells by SARS-CoV-2 Vero

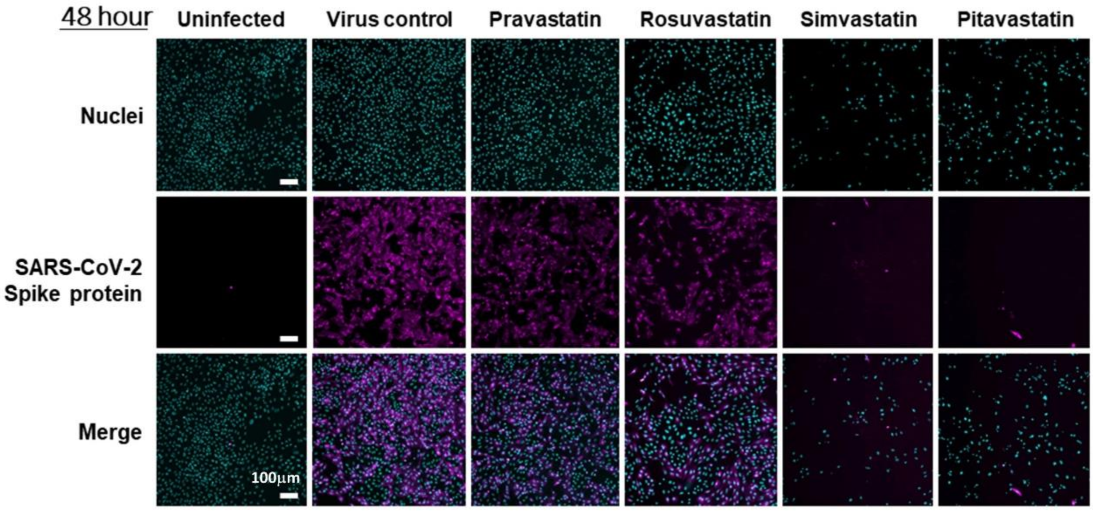

Figure S7. Statins do not reduce infection of Vero cells by SARS-CoV-2 Vero

Vero cells were plated in 96 well plates at $8x10^3$ cells/well for 24 hours before inoculating with SARS-CoV-2 strain hCOV-19/England/2/2020 at 167 IU/well with 100nM statins or DMSO equivalent in a total volume of 50ml. After 48 hours, supernatant was removed and cells were fixed in ice-cold methanol. Cells were then stained with rabbit anti-SARS-COV-2 spike protein, subunit 1 followed by Alexa Fluor 555-conjugated goat anti-rabbit IgG secondary antibody. Cell nuclei were visualised with Hoechst 33342. Cells were washed with PBS and then imaged and analysed using a Thermo Scientific CellInsight CX5 High-Content Screening (HCS) platform. Infected cells were scored by perinuclear fluorescence above a set threshold determined by positive (untreated) and negative (uninfected) controls. A minimum of 9 fields and 5000 nuclei per well in triplicate or quadruplicate wells per treatment were scored in each experiment. All experiments were n=2-4 with 3-4 repeats in each experiment.

Figure S8. Statins do not reduce infection of Vero cells with SARS-CoV-2

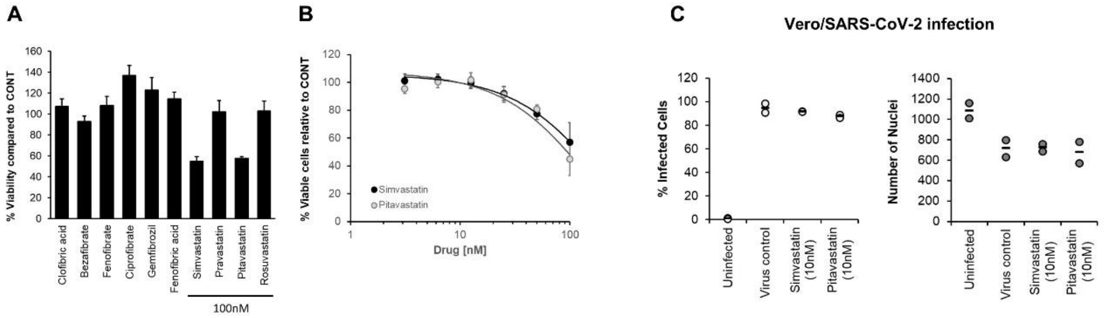

Figure S8. Statins do not reduce infection of Vero cells with SARS-CoV-2

Vero cells were plated in 96 well plates at 8x10^3 cells/well for 24 hours before treating with either 100nM statins (A) or doubling dilutions of simvastatin or pitavastatin (B) in 200ul. Viability was assessed at 48 hours using Cell-titre Blue (Promega) assay and viability calculated relative to DMSO controls. (C) Vero cells were infected with SARS-CoV-2 strain hCOV-19/England/2/2020 at 167 IU/well with 10nM statins in a total volume of 50ml. After 48 hours, supernatant was removed and cells were fixed in ice-cold methanol. Cells were then stained with rabbit anti-SARS-COV-2 spike protein, subunit 1 followed by Alexa Fluor 555-conjugated goat anti-rabbit IgG secondary antibody. Cell nuclei were visualised with Hoechst 33342. Cells were washed with PBS and then imaged and analysed using a Thermo Scientific CellInsight CX5 High-Content Screening (HCS) platform. Infected cells were scored by perinuclear fluorescence above a set threshold determined by positive (untreated) and negative (uninfected) controls. A minimum of 9 fields and 5000 nuclei per well in triplicate or quadruplicate wells per treatment were scored in each experiment. All experiments were performed n=2-4 with 3-4 repeats in each experiment.
